# Management of post-operative pancreatic fistulas following Longmire–Traverso pylorus-preserving pancreatoduodenectomy by endoscopic vacuum-assisted closure therapy

**DOI:** 10.1186/s12876-021-02000-3

**Published:** 2021-11-12

**Authors:** Dominik J. Kaczmarek, Dominik J. Heling, Maria A. Gonzalez-Carmona, Christian P. Strassburg, Vittorio Branchi, Hanno Matthaei, Jörg Kalff, Steffen Manekeller, Tim R. Glowka, Tobias J. Weismüller

**Affiliations:** 1 Department of Internal Medicine I, University of Bonn, Bonn, Germany; 2 Department for General, Visceral, Thoracic and Vascular Surgery, University of Bonn, Bonn, Germany; 3 Present Address: Department of Internal Medicine - Gastroenterology and Oncology, Vivantes Humboldt Hospital, Am Nordgraben 2, 13509 Berlin, Germany

**Keywords:** Endo-sponge, Endoscopic vacuum-assisted wound closure system, Anastomotic leakage, Pancreaticogastric anastomotic insufficiency, Pancreaticogastrostomy

## Abstract

**Background:**

Pylorus-preserving pancreatoduodenectomy (PPPD) with pancreatogastrostomy is a standard surgical procedure for pancreatic head tumors, duodenal tumors and distal cholangiocarcinomas. Post-operative pancreatic fistulas (POPF) are a major complication causing relevant morbidity and mortality. Endoscopic vacuum therapy (EVT) has become a widely used method for the treatment of intestinal perforations and leakages. Here we report on a pilot single center series of 8 POPF cases specifically caused by dehiscences of the pancreatogastric anastomosis (PGD), successfully managed by EVT.

**Methods:**

We included all patients with PGD after PPPD, who were treated with EVT between 07/2017 and 08/2020. For EVT a vacuum drainage film (EVT film) or open-pore polyurethane foam sponge (EVT sponge) was fixed to a 14Fr or 16Fr suction catheter and placed endoscopically within the PGD for intracavitary EVT with continuous suction between − 100 and − 150 mmHg. The EVT film/sponge was exchanged twice per week. EVT was discontinued when the PGD was sufficiently healed.

**Results:**

PGD closure was achieved in 7 of 8 patients after a mean EVT time of 16 days (range 8–38) and 3 EVT film/sponge exchanges (range 1–9). One patient died on day 18 after PPPD from acute hemorrhagic shock, unlikely related to EVT, before effectiveness of EVT could be fully achieved. There were no adverse events directly attributable to EVT.

**Conclusions:**

EVT could be an effective and safe addition to our therapeutic armamentarium in the management of POPF with PGD. Unless prospective comparative studies are available, EVT as minimally invasive therapeutic alternative should be considered individually by an interdisciplinary team involving endoscopists, surgeons and radiologists.

## Background

The surgical treatment for neoplastic or preneoplastic disorders of the pancreatic head, the distal bile duct and the major duodenal papilla is a pancreatoduodenectomy. The standard surgical procedures are the classic pancreatoduodenectomy (Kausch–Whipple) with pancreatojejunostomy, or the pylorus-preserving pancreatoduodenectomy (PPPD) with the Traverso–Longmire method [1, 2]. The PPPD can be either combined with a pancreatojejunostomy or a pancreatogastrostomy. Studies comparing both methods report conflicting results [3–5]. Pancreatoduodenectomy is a complex procedure associated with mortality rates up to 3% in recent series and a variety of possible post-operative complications, including delayed gastric emptying (mean incidence of 17%), bile leak from the choledochal-jejunal anastomosis in 1–3% and new-onset diabetes in 16–22% of cases [3, 5–12]. Another relevant complication is the development of post-operative pancreatic fistulas (POPF). POPF are the major cause of morbidity after pancreatic resection, affecting up to 41% of cases and carrying high mortality rates up to 28% [3, 7–9, 13–16]. POPF severity is classified into “biochemical leak” (“BL”, formerly grade A), grade B and grade C according to the International Study Group on Pancreatic Surgery (ISGPS) [17, 18]. POPF can be the result of a parenchymal leak into the peripancreatic/retroperitoneal region or manifest as dehiscence of a pancreatic-enteric anastomosis [17]. Drain placement during surgery and post-operative monitoring of drainage color, drainage amount and amylase concentration help to detect a POPF [19]. In some cases of asymptomatic amylase-rich drain effluent (grade A fistula), POPF may heal without intervention [9, 20]. But since POPF can lead to sepsis and hemorrhage [3, 9, 21], urgent therapy is mandatory in symptomatic patients with fever, pain or rising inflammation parameters (grade B and C). The therapeutic arsenal contains interventions such as percutaneous drainage, endoscopic ultrasound-(EUS)-guided transenteric drainage or revision surgery.

The successful use of endoscopic vacuum therapy for POPF in cases where they become manifest as dehiscence of the pancreatogastric anastomosis (PGD) has been described in two case reports [22, 23]. Endoscopic vacuum therapy (EVT), also referred to as endoscopic vacuum-assisted closure (endo-VAC) therapy, has become a standard treatment of perforations, fistula formation or anastomotic insufficiencies mainly in the upper gastrointestinal (GI) tract or the rectum since it was first described by Weidenhagen 2003 [23–29]. This method is derived from vacuum-assisted closure (VAC) therapy of external wounds, as introduced by Argenta and Morykwas in 1997 [30]. EVT within the GI tract involves a polyurethane foam sponge or a special open-pore film, connected to a suction catheter, which is endoscopically placed either into the lumen of the GI tract, thus covering the perforation site (intraluminal EVT), or inserted into the perforation site itself, i.e. outside of the mucosa-lined space (intracavitary EVT) [25, 31]. While VAC systems for endoscopic use formerly had to be shaped and assembled individually by the endoscopist, ready-to-go EVT systems are nowadays commercially available.

In this study, we report on our pilot series of POPF patients after PPPD who specifically manifested as pancreatogastric dehiscence (POPF with PGD) and who were treated with EVT.

## Methods

### Patient cohort

For this retrospective analysis, we included all consecutive patients from our institution diagnosed with a POPF after PPPD between July 2017 and August 2020, who had specifically developed a PGD as underlying cause of the POPF. Of note, we defined PGD as clinically apparent continuity defect (insufficiency) of the circular pancreatogastric anastomosis, as seen endoscopically. Such continuity defects permit a leakage of gastric contents (including gastric and pancreatic fluids) into the retroperitoneum, thus promoting the formation of a retroperitoneal wound cavity. To identify patients, we performed a systematic data base search within our endoscopy documentation system (Viewpoint, General Electric). All PPPD were performed by the Department for General, Visceral, Thoracic and Vascular Surgery of the University Hospital Bonn. POPF detection and grading was according to ISGPS. Briefly, amylase activity was measured in the intraoperatively placed wound drains on post-operative day 3 and considered suspect if amylase content was greater than 3 times the upper normal serum value [17, 18]. Moreover, the drains were monitored for increasing fluid amounts, changing color and bilirubin concentration. If a POPF was suspected measurement of amylase activity was repeated and computed tomography (CT) scans for further assessment were performed. Radiological and clinical findings were then used for the grading of the POPF. All patients with POPF grade B and C were treated. In case of abdominal fluid collections, transenteric drainage was the preferred treatment. If this was not possible (e.g. fluid collection located in the lower abdomen), a transcutaneous CT-guided drain was placed. If a PGD was suspected, patients received upper GI tract endoscopy and EVT was the treatment of choice. Outcome measures were successful closure of the PGD and healing of the associated retrogastral wound cavity, endoscopy-associated complications, mortality and the persistence or recurrence of a PGD/POPF despite endoscopic therapy.

### Endoscopic vacuum therapy

**Fig. 1:**
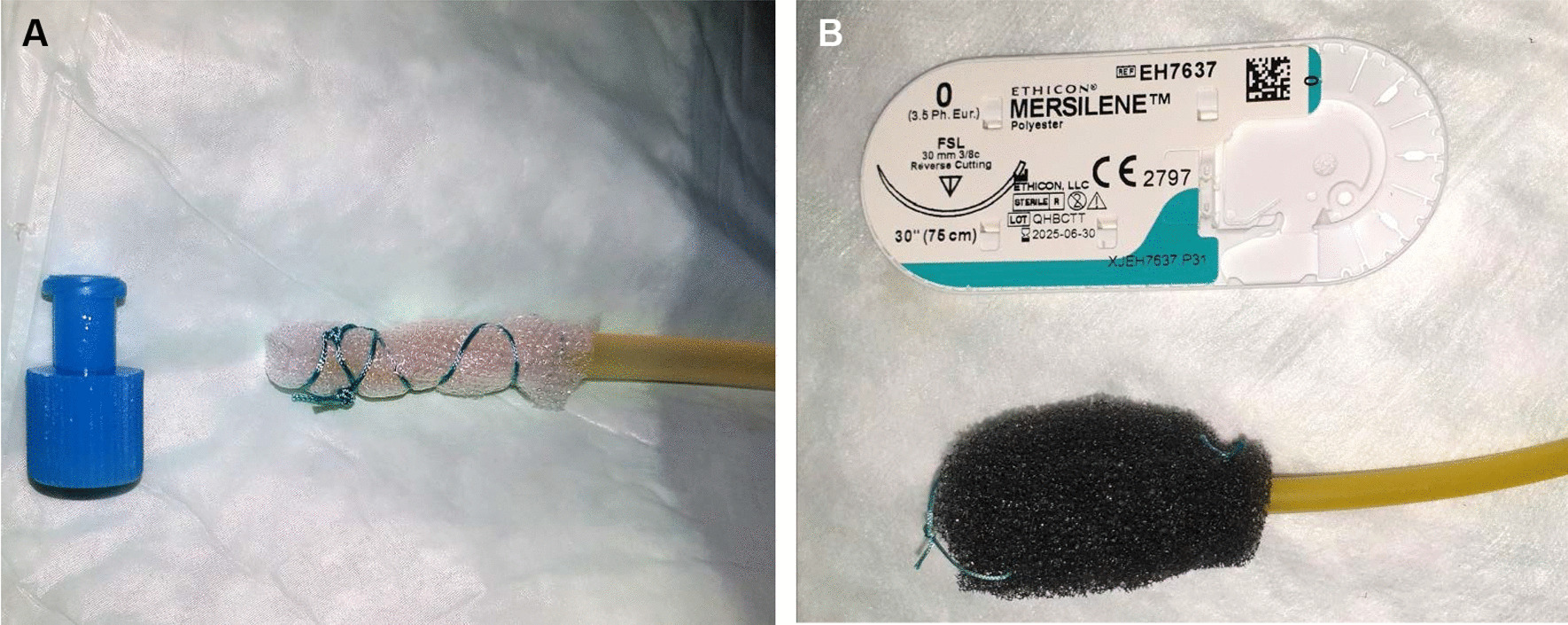
Depending on the size of the pancreatogastric dehiscence (PGD), either an open-pore film **(A)** or an open-pore polyurethane foam sponge **(B)** is individually shaped by the endoscopist to fit into the PGD and attached to the tip of a 14Fr or 16Fr suction catheter by sutures. The film/sponge is then placed into the PGD. The other end of the catheter is connected to a vacuum pump which applies continuous suction to the PGD and its associated retrogastral wound cavity. Closing cone and suture package serve as size reference

EVT was performed according to previous reports [25, 31]. In detail, a single-lumen 14Fr or 16Fr suction catheter was transnasally inserted and perorally retrieved. Depending on the PGD size either a thin vacuum drainage film (EVT film) (Suprasorb-CNP, Lohmann & Rauscher, Neuwied, Germany) or an open-pore polyurethane foam sponge (EVT sponge) (V.A.C. Granufoam Dressing Medium, Acelity, Wiesbaden, Germany) was individually shaped by the endoscopist to fit into the PGD and attached to the tip of the suction catheter (EVT catheter) by sutures (Mersilene Polyester, 0/3.5Ph.Eur., Ethicon) as shown in Fig. 1. If necessary, additional suction holes were cut into the catheter to distribute suction to the EVT film/sponge at its full length. Due to large variations in PGD shape among patients, we decided to individually shape the EVT sponge according to our needs rather than deploy a readily manufactured sponge system, as commercially available for EVT. In case of EVT films, there are no commercially available ready-to-use devices, so these must always be shaped individually by the endoscopist. The EVT film/sponge was then gripped with a rat tooth grasping forceps (2.3 mm diameter, Endo-Flex, Voerde, Germany), endoscopically guided into the stomach (GIF-1TH190, Olympus, or EG-530CT, Fujifilm) and then inserted into the PGD under endoscopic view for intracavitary EVT. The EVT catheter was connected to a vacuum pump (V.A.C. VeraFlo, v.a.c.ulta, Negative Pressure Wound Therapy Unit, Dublin, Ireland) with continuous suction. Suction intensity (negative pressure between 100 and 150 mmHg) was chosen at the endoscopist’s discretion following their experience. Once firm position of the EVT film/sponge within the PGD under suction was confirmed, the endoscope was retrieved and the EVT catheter fixed to the nose by a tape. In some cases, an additional triple lumen naso-gastral/naso-jejunal feeding tube (Freka Trelumina Fr16/9, Fresenius Kabi, Bad Homburg, Germany) was transnasally inserted and positioned in the jejunum. The EVT film/sponge was exchanged twice per week. For EVT exchange, correct position of the inserted EVT film/sponge was endoscopically confirmed. Suction was then discontinued and the EVT film/sponge perorally removed with a rat tooth grasping forceps. The PGD was inspected and assessed for size; if necessary, the cavity was cleaned of debris, fibrin and necrotic tissue and rinsed with NaCl 0.9%. A new EVT film/sponge was then attached to the suction catheter and reinserted into the PGD as described above. EVT was discontinued as soon as the PGD-associated cavity displayed no debris and was covered with granulation tissue, there were no signs of active cavity-feeding fistulas and the cavity had become too small to insert a new EVT film/sponge in a reasonable manner. All endoscopic procedures were performed by a team of experienced interventional gastroenterologists either under general anesthesia or conscious sedation with midazolam and/or propofol depending on patients’ current health state.

### Data presentation

Data are presented as mean values with range or, where applicable, as percentage of all patients.

## Results

### Patient characteristics and diagnosis of PI

We identified 8 patients with a POPF grade B or C after PPPD who had specifically developed a PGD as underlying cause of the POPF. Patient characteristics are given in Table 1. All patients were male and the mean age at PPPD was 56 years (range 37–79). Indications for pancreatectomy were neoplastic disorders in 6 cases (75%). In one case PPPD was performed due to chronic pancreatitis with recurring acute episodes and with an inflammatory pseudotumor of the pancreatic head. Another patient with chronic pancreatitis had a complicated pseudocyst within the pancreatic head. All patients received percutaneous wound drains post-operatively (Silicone Penrose drains, Fortune Medical Instrument Corp., New Taipeh City, Taiwan). A POPF was first assumed after a mean period of 13 days following PPPD (range 2–28) (Table 2). All patients were scheduled for upper GI tract endoscopy because a PGD as underlying cause of the POPF was suspected and further therapy urgent since percutaneous wound drainage alone did not suffice to improve the patient’s health state. The mean size of the PGD in all 8 patients during first endoscopy was 8 mm (range 2–15).

**Table 1:** Patient characteristics and indication for pancreatectomy

Patient no	Age (years)	Sex (m/f)	Indication for pancreatectomy
1	60	m	Distal bile duct cancer (cholangiocarcinoma)
2	49	m	Distal bile duct cancer (cholangiocarcinoma)
3	37	m	Chronic pancreatitis with inflammatory pseudotumor of the head
4	54	m	Neuroendocrine tumor (NET) of the duodenum
5	51	m	Neuroendocrine tumor (NET) of the pancreas
6	64	m	Chronic pancreatitis with complicated pseudocyst
7	79	m	Adenocarcinoma of the pancreatic head
8	57	m	Carcinoma of the major duodenal papilla (papillary carcinoma)

All patients underwent pylorus-preserving pancreatoduodenectomy (PPPD) with pancreatogastrostomy

**Table 2:** Synopsis of basic endoscopic vacuum therapy (EVT) data

Patient no	POPF grade (ISGPS A/B/C)	POPF diagnosis (days post PPPD)	Initial PGD size (mm)	Begin EVT (days post PPPD)	Initial EVT system (film/sponge)	No. of EVT film/sponge exchanges	Duration of continuous EVT (days)	Additional endoscopic therapy	Initial EVT pressure (mmHg)	Successful PGD closure at the end of EVT	Outcome
1	B	15	8	24	Sponge	6	29	PGD dilation (10 mm balloon, single session) and DEN	125	Yes	Died 13 months post PPPD from relapse of cholangiocarcinoma
2	B	11	10	12	Film	1	8		125	Yes	Alive until follow-up
3	B	3	8	13	Sponge (later film)	2	8		125	Yes	Alive until follow-up
4	C	28	4	33	Film	2	11		125	Yes	Alive until follow-up
5	B	22	8	26	Film	2	11		125	Yes	Alive until follow-up
6	B	2	12	2	Sponge	4	14		100 (later increased to 125)	Yes	Alive until follow-up
7	B	17	15	18 (2nd cycle: 63)	Sponge	6 (2nd cycle: 3)	23 (2nd cycle: 15)	DEN and 22 days EVT-free interval between 1st and 2nd EVT cycle with transgastral double pig-tailed drainage of wound cavity	125 (later increased to 150)	Yes (after 2nd cycle)	Died 5 months post PPPD from pneumoseptic and uroseptic shock
8	C	6	2	12	Film	1	6		100	No	Died 1 month post PPPD from hemorrhagic shock before proof of effectiveness of EVT

DEN (direct endoscopic necrosectomy), ISGPS (International Study Group on Pancreatic Surgery), PGD (pancreatogastric dehiscence), POPF (post-operative pancreatic fistula), PPPD (pylorus-preserving pancreatoduodenectomy)

### Endoscopic vacuum therapy

**Fig. 2:**
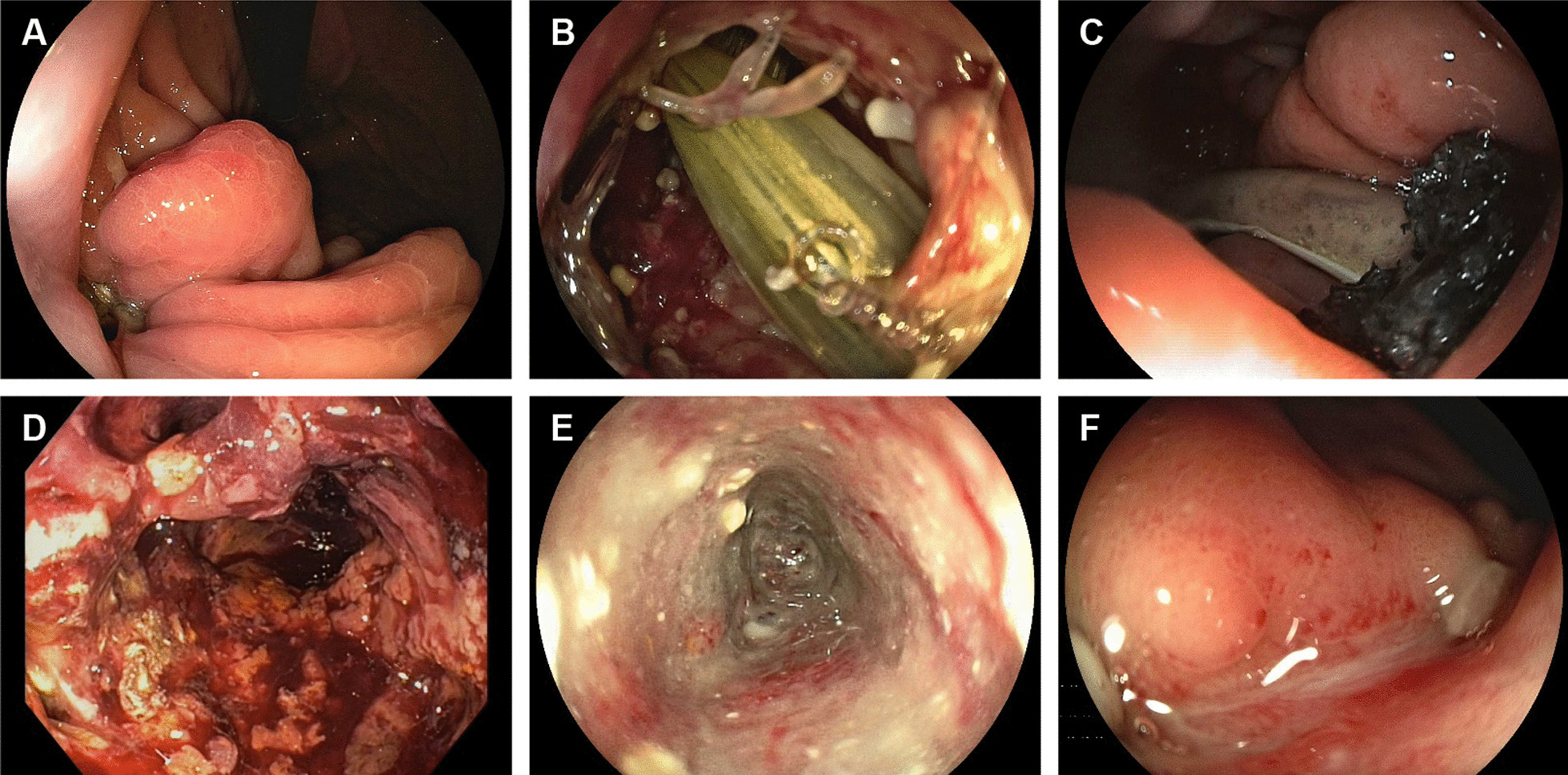
Endoscopic view of the stomach with a post-operative pancreatogastric dehiscence (PGD). All pictures refer to the same patient (“patient no. 1”). **A** PGD at first endoscopic encounter (day 0). **B** View through the PGD into the PGD-associated retrogastral wound cavity containing percutaneous wound drainage (day 0). **C** Intracavitary position of a polyurethane foam sponge. A vacuum pump applies continuous suction to the sponge through a 16Fr catheter. The percutaneous wound drainage has been partially withdrawn to not interfere with the endoscopic vacuum therapy (EVT). **D** Ongoing EVT unmasked a large retrogastral necrotic wound cavity (day 15 of EVT). The PGD channel had to be dilated with a 10 mm dilation balloon four days earlier to permit direct endoscopic necrosectomy (DEN) and facilitate sufficient insertion of the sponge into the cavity. **E** Wound cavity on the last day of continuous EVT. EVT was successfully ended after 29 days. **F** Entirely closed PGD seven weeks later

All 8 patients received EVT to treat the PGD. Basic EVT data are summarized in Table 2. The mean time between first clinical signs of a POPF and beginning of EVT was 5 days (range 0–10). In 4 patients EVT was initiated with an EVT film (50%) and in 4 patients with an EVT sponge (50%). In all patients the EVT film/sponge was inserted directly into the PGD without necessity of prior dilation of the wound channel. In one patient (“patient 1”, Tables 1 and 2) with an initial PGD size of 8 mm, however, ongoing EVT unmasked a larger retrogastral necrotic wound cavity, so the endoscopist decided to dilate the PGD with a 10 mm dilation balloon (single dilation session, Fusion Titan Biliary Dilation Balloon, Cook Medical, Ireland) to allow for necrosectomy and facilitate sufficient insertion of the EVT sponge into the cavity (Fig. 2). In five patients a negative pressure of − 125 mmHg and in one patient of − 100 mmHg was applied for the entire EVT (at medium intensity). In two patients, the endoscopist decided to increase the negative pressure during the course of therapy from − 100 to − 125 mmHg and from − 125 to − 150 mmHg, respectively, because induction of granulation tissue was insufficient. In six patients (“patients 1–6”, Tables 1 and 2) healing of the PGD by EVT could be achieved after a mean period of 14 days (range 8–29) and 3 EVT film/sponge exchanges (range 1–6).

**Fig. 3:**
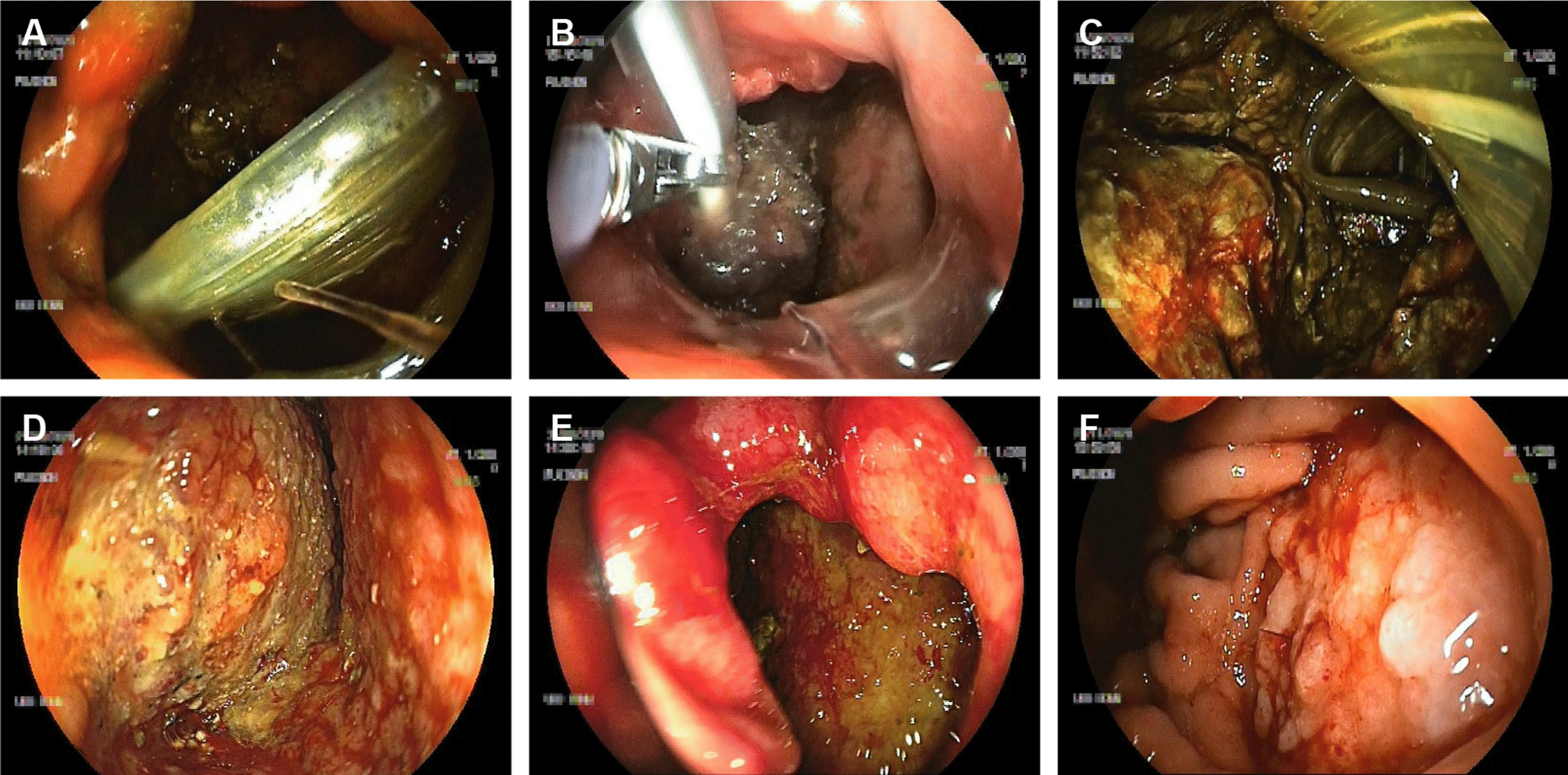
Endoscopic view of the stomach with a post-operative pancreatogastric dehiscence (PGD). All pictures refer to the same patient (“patient no. 7”). **A** Large PGD orifice, approximately 15 mm in diameter, with view into the PGD-associated retrogastral wound cavity on first endoscopic encounter (day 0). The remaining pancreas is completely torn off the stomach wall. A percutaneous wound drainage is visible within the cavity. **B** The endoscopist inserts a polyurethane foam sponge into the cavity by a rat tooth grasping forceps. Endoscopic vacuum therapy (EVT) is then established by a vacuum pump which applies continuous suction to the sponge through a 16Fr catheter. **C** Partial view over the large retrogastral necrotic wound cavity containing percutaneous wound drainages (day 3 of EVT). **D** Wound cavity on day 16 of ongoing EVT and after direct endoscopic necrosectomy (DEN). Granulation tissue can be seen on the cavity walls. **E** PGD orifice with view into the wound cavity on day 20 of ongoing EVT. A first cycle of EVT (23 days) did not achieve effective reduction in cavity size. After discontinuation of EVT a second EVT cycle was launched 22 days later. Following another 15 days of EVT, the cavity size was eventually sufficiently small to discontinue EVT. **F** Complete closure of the PGD six weeks later

One patient (“patient 7”, Tables 1 and 2) developed a severe post-operative hemorrhage from the lienal artery one day after PPPD and had to undergo revision surgery with reattachment of the remaining pancreas to the stomach. On day 10 after PPPD the patient underwent another revision surgery because of superinfected hematoma. 17 days after PPPD drainage fluid color suggested a GI tract leakage and on day 18 an upper GI tract endoscopy revealed a PGD with a round-shaped perforation orifice of 15 mm diameter (Fig. 3a, b). The remaining pancreas was completely torn off the stomach and a large retrogastral cavity could be seen endoscopically (Fig. 3c). A first cycle of EVT (23 days, 6 EVT sponge exchanges) induced plenty formation of granulation tissue within the cavity but could not achieve an effective reduction in cavity size (Fig. 3d–e). Therefore, EVT was discontinued and the cavity drained through multiple transgastral double pig-tailed stents. 22 days later all percutaneous drainages and transgastral stents were removed and a second EVT cycle was launched. The POPF orifice diameter in the stomach was 30 mm by that time. Following another 15 days of EVT and 3 EVT sponge exchanges, the size of the retrogastral cavity markedly decreased and EVT was successfully discontinued. Complete closure was confirmed 6 weeks later (Fig. 3f).

Another patient (“patient 8”, Tables 1 and 2) died 18 days after PPPD from acute retrogastral hemorrhage while EVT was still ongoing (day 6 of EVT, negative pressure 100 mmHg, one exchange of EVT film up to that time). There were no signs of upper GI tract bleeding.

All EVT patients (100%) were initially kept on nothing per os (NPO) and instead received complete parenteral nutrition intravenously. In two patients (25%) a triple lumen naso-gastral/naso-jejunal tube (Freka Trelumina Fr16/9, Fresenius Kabi, Bad Homburg, Germany) was inserted into the jejunum during ongoing EVT for enteral nutrition and for drainage of gastric fluid. Two patients (25%) had a naso-gastral tube for drainage of gastric fluid only.

### Outcome

Eventually, POPF healing by EVT could be achieved in 7 of 8 patients (88%). The eighth patient (“patient 8”, Tables 1 and 2) died from acute hemorrhagic shock on day 18 after PPPD while EVT was still ongoing. The bleeding manifested at the percutaneous wound drains and was fulminant: The patient was immediately transferred to our angiography room for intended vascular intervention but died before angiography could be started despite ongoing resuscitation measures. A POPF-associated bleeding from the operation site was considered the most likely reason for the hemorrhagic shock. A postmortem examination was rejected by the patient’s family. Of note, there were no signs of upper GI tract bleeding and therefore an association between the bleeding and EVT itself seemed unlikely. However, a causative association cannot be excluded. Apart from that, no other EVT-associated adverse events were recorded among all patients. None of the patients had to undergo surgical revision of the POPF, once EVT was initiated, and there were no cases of recurrent POPF/PGD, once EVT was successfully completed. The mean follow-up time for all seven patients who had been successfully discharged from EVT was 5 months (range 1–11). One patient died from pneumoseptic and uroseptic shock 5 months after PPPD (“patient 7”, Tables 1 and 2) and one patient died from recurrence of distal cholangiocarcinoma 13 months after PPPD (“patient 1”, Tables 1 and 2). The other five patients survived until last follow-up.

## Discussion

Post-operative pancreatic fistulas (POPF) affect up to 41% of cases and are a major driver of morbidity and mortality following pancreatic resection [3, 7–9, 13–15]. EVT has become a standard procedure for treatment of gastrointestinal leakages such as traumatic or iatrogenic perforations or post-operative anastomotic leakages within the esophagus, stomach, duodenum or rectum [23, 25–29]. In a case report by Schorsch et al. in 2013, the authors describe the successful use of EVT to treat a PGD [22]. For this purpose, the authors inserted a large sponge into the stomach covering the PGD and aiming at continuous deflation/drainage of the stomach. In 2015 Borejsza-Wysocki et al. also reported on a case of effective use of EVT for a PGD [23]. These two case reports suggest that patients with PGD following PPPD could analogically benefit from EVT, thus reducing the need for further surgical high-risk interventions [22, 23, 32].

In this present retrospective pilot study, we confirm the usefulness of EVT for clinically relevant PGD following PPPD (POPF grade B and C). Since first establishment of EVT as therapeutic strategy for PGD in our endoscopy unit (July 2017), EVT could induce a sustained healing of the POPF/PGD and avoid further surgical interventions in seven of eight patients (88%). One patient (13%) died 18 days after PPPD from fulminant hemorrhagic shock while EVT was still ongoing and its full therapeutic impact on PGD closure had not yet been achieved. EVT can cause erosion bleedings so a causative impact of EVT on the hemorrhage must be taken into consideration. On the other hand, the dehiscence at the pancreatic anastomosis was very distinct in this case (2 mm in length), so we had only used a short and narrow EVT film with a reduced suction rate (100 mmHg). This as well as the fact that blood came from the percutaneous wound drain but neither from the EVT catheter itself nor from the nasogastric drain argue against EVT as leading cause of the hemorrhage. Also, high bleeding and mortality rates among PPPD patients with POPF are common and expectable [3, 7–9, 14, 15, 32]. Apart from that, no other EVT-associated adverse events were recorded in our study.

Due to its retrospective design without a matched control cohort our study has some inevitable limitations that make it difficult to generalize our results. A selection bias cannot be fully excluded: although all patients with POPF grade B/C and suspected PGD as underlying cause were introduced to upper GI tract endoscopy and thus eligible for EVT, presence of PGD in all other patients with POPF (but no suspicion of PGD) cannot be fully excluded. Similarly, it can be speculated that some of the PGD patients might have also recovered without EVT. On the other hand, all patients treated in this study had clinically relevant POPF (grade B or C) and initiation of a treatment was mandatory since recovery without further specific therapy (other than percutaneous drainage) was considered unlikely. Moreover, we did not compare efficacy and safety of EVT to non-endoscopic forms of management, like surgical, radiological or conservative approaches (“watch and wait”), and we did not compare EVT with other endoscopic closure techniques, like clip application. However, studies focusing on other parts of the GI tract, e.g. the esophagus or the rectum, confirm that EVT is an effective and safe method to treat leakages [33–36]. Several meta-analyses found that EVT in comparison to the use of self-expandable metal stents (SEMS) for esophageal leaks had higher closure rates, shorter treatment times and lower mortality rates [37, 38]. Kühn et al. compared EVT to conventional treatment for leakages after rectal resection, like percutaneous drainage and relaparotomy, and conclude that EVT might be more effective in terms of definite healing and preserving of intestinal continuity [35].

The standard management of POPF comprises interventional techniques, like image-guided percutaneous or EUS-guided transenteral drainage of peripancreatic fluid collections [16, 39]. A high percentage of post-pancreatectomy patients (more than 85%) can be successfully managed with percutaneous drainage without the need for revision surgery [16, 40, 41]. In this context, EUS-guided drainage seems to be equally effective with technical success rates between 90 and 100% and treatment success rates between 79 and 100% [16, 42–45]. Patients who fail to respond to interventional techniques (about 20% of POPF patients depending on study), however, require a surgical re-intervention, e.g. reconstruction of the pancreatic-gastric anastomosis or even complete pancreatectomy [16]. Studies comparing EVT to other forms of POPF treatment, as mentioned above, are not available yet. Importantly, EVT in case of POPF addresses a specific subset of patients only who explicitly present with a dehiscence of the pancreatogastric anastomosis. This as well as the fact that EVT is often used simultaneously with our ongoing therapies (like percutaneous drainage) makes it difficult to compare EVT to other forms of POPF treatment.

As a pilot study that primarily shows the feasibility and effectiveness of EVT in PGD patients, our study leaves some relevant questions unanswered: (1) Which patients are promising candidates for EVT and which patients will not benefit from EVT in the setting of a PGD? Further prospective multicenter studies are needed to establish reliable criteria for identification of candidates for EVT. In our study, PGD size neither seems to serve as an appropriate criterion for EVT initiation nor as a good predictor of EVT duration (i.e. time until PGD closure is achieved). (2) What is the optimal time-point to discontinue EVT? It is unclear if the PGD-associated wound cavity must be treated until it is maximally downsized (as in our study) or if EVT can be safely discontinued at an earlier stage. (3) In our case series, we have inserted the EVT film/sponge into the PGD itself. In 2013 Schorsch et al. reported on a case where they had successfully treated a PGD by intraluminal EVT with placement of a large sponge into the gastric lumen, thus covering the PGD [22]. Placing the EVT film/sponge into the PGD can be cumbersome and there is a relevant risk of VAC film/sponge dislocation back into the stomach, so comparing studies would be helpful to elicit whether intracavitary EVT is superior to intraluminal EVT. (4) Furthermore, re-establishment of enteral nutrition (via naso-jejunal tube or feeding jejunostomy) is considered an important step in the standard management of clinically relevant POPF [16]. Enteral nutrition increases the probability of fistula closure, shortens time to closure and is associated with faster recovery [46]. Some of our patients in fact received naso-jejunal tubes during ongoing EVT to allow for enteral nutrition. However, in our experience placement of naso-intestinal tubes during ongoing EVT is cumbersome and poses the risk of interference with the VAC system leading to its dislocation. Thus, prospective studies paying more attention to the use of naso-jejunal tubes are needed and might help to determine their role during ongoing EVT.

## Conclusions

We present a proof-of-principle study showing that intracavitary EVT is a safe and effective minimally invasive approach for the management of clinically relevant PGD following PPPD. These results might justify further prospective multicenter studies in order to confirm our findings, to better characterize the optimal treatment strategies and EVT modalities for patients with PGD. Unless prospective comparative studies are available, EVT as minimally invasive therapeutic alternative should be considered individually by an interdisciplinary team involving endoscopists, surgeons and radiologists.

## Data Availability

All data generated or analyzed during this study are included in this published article. The datasets used and analyzed during the current study are also available from the corresponding author on reasonable request (Tobias J. Weismüller, email: tobias.weismueller@gmx.de).

## Abbreviations

BL: Biochemical leak
CT: Computed tomography
DEN: Direct endoscopic necrosectomy
endo-VAC: Endoscopic vacuum-assisted closure
EUS: Endoscopic ultrasound
EVT: Endoscopic vacuum therapy
GI: Gastrointestinal
ISGPS: International Study Group on Pancreatic Surgery
NPO: Nothing per os
PGD: Pancreatogastric dehiscence
POPF: Post-operative pancreatic fistula
PPPD: Pylorus-preserving pancreatoduodenectomy
SEMS: Self-expanding metal stent
VAC: Vacuum-assisted closure
